# Factors associated with improved outcomes after lumbar transforaminal epidural steroid injections for radicular pain: A systematic review

**DOI:** 10.1016/j.xnsj.2025.100592

**Published:** 2025-01-28

**Authors:** Minjin K. Fromm, Shao-Hsien Liu, Catherine Carr, Elizabeth Stefancic, Michael Rogalski

**Affiliations:** Department of Orthopedics and Physical Rehabilitation, UMass Chan Medical School, Worcester, MA, United States

**Keywords:** Spinal and epidural injections, Sciatica, Radiculopathy, Back pain, Intervertebral disc displacement and degeneration, Spinal stenosis, Spondylosis, Prognosis, Predictors

## Abstract

**Purpose:**

Despite numerous studies, the factors contributing to clinical success after a lumbar transforaminal epidural steroid injection (LTFESI) for radicular pain remain unclear. The aim of this study was to systematically review literature evaluating preprocedural predictive factors for improved outcomes in patients receiving a LTFESI.

**Methods:**

We searched databases including PubMed/MEDLINE, Cochrane Library, and Scopus for studies published from 2006 through 2023. The Preferred Reporting Items for Systematic Reviews and Meta-Analyses (PRISMA) guidelines were followed to identify articles evaluating prognostic factors associated with outcomes after a LTFESI. Studies were excluded if articles treated patients with axial back pain or if they were not performed under imaging guidance with contrast medium. The quality of included studies was appraised by 2 reviewers using the Quality in Prognosis Studies tool (QUIPS).

**Results:**

Eighteen studies met eligibility criteria which evaluated prognostic factors across domains including patient characteristics, clinical findings, magnetic resonance imaging (MRI) characteristics, anatomical variations, and electromyography findings. The largest predictive factor studied were the MRI findings causing radicular pain. A lower grade of nerve compression and a lumbar disc herniation which was central, foraminal or extraforaminal was associated with improved outcomes, as opposed to a subarticular location. Lower paraspinal mapping scores on electromyography were correlated with better outcomes than higher scores. Outcomes were not affected if a lumbar disc herniation was accompanied by degenerative changes or if the cause of radicular pain was from a lumbar disc herniation or from lumbar spinal stenosis. The risk of bias across many domains in the studies were varied being highest overall in the domains of study attrition and study confounding.

**Conclusions:**

The evidence behind factors that predict outcomes from a lumbar transforaminal epidural steroid injection have varying degrees of bias, but trends appear that can be used to guide clinical decision making.

## Introduction

Lumbar radicular pain is buttock and/or leg pain referred from the low back, thought to be caused by an interplay of inflammatory, immunological and pressure related processes on the nerve roots. Most causes are degenerative such as from lumbar disc herniations and lumbar spinal stenosis, with other causes such as malignancy and infection occurring less frequently [1]. Epidemiological studies for sciatica, which is a form of lumbar radicular pain, has shown a lifetime prevalence ranging from 1.2% to 43% [1] and an annual incidence of 1-5% [2].

The usual care involves activity modification, medications, exercise-based treatment, and steroid injections with surgery offered for patients who do not have resolution of symptoms. Lumbar epidural steroid injections can help radicular pain by delivering medications close to the nerve root where steroids act locally through its anti-inflammatory effects, including inhibition of the formation of enzymes such as cyclo-oxygenase and lipoxygenase that contribute to prostaglandin formation, as well as the protection of C- fibers to decrease pain [3]. Typically, lumbar epidural steroid injections can be performed via 3 routes: interlaminar, caudal and transforaminal. When comparing the 3 routes of administration, studies have shown that the transforaminal route can be more effective in relieving radicular pain caused by disc herniation, presumably due to delivery of steroid into the anterior epidural space, closer to the site of nerve root inflammation [[4], [5], [6]]. A study of utilization patterns of epidural steroid injections in the Medicare population showed a significant shift from 2000 to 2018, in favor of more transforaminal injections being performed compared with the interlaminar approach. In 2018, lumbar transforaminal epidural steroid injections made up 53% of all lumbar epidurals performed, compared to 14.6% in 2000. [7]

Despite its routine use for lumbar radicular pain, the response to lumbar transforaminal epidural steroid injections (LTFESIs) differs among individuals and remains controversial in the medical literature. It is suggested that the typical response to LTFESIs ranges broadly from no relief to continued relief beyond 12 months, with minimal to 100% improvement of symptoms [8]. Many studies have evaluated prognostic factors for improved outcomes after LTFESIs for radicular pain. However, a systematic review regarding the prognostic factors associated with outcomes after LTFESI is still lacking. To address this knowledge gap, we conducted a systematic review to provide an in-depth summary of recent literature that evaluated preprocedural prognostic factors and their relationship to outcomes.

## Methods

The research included studies that were obtained from publicly available sources and therefore the study was not considered human subject research.

### Search Strategy

This study followed the guidelines set forth by the PRISMA statement [9]. A comprehensive literature search was conducted by a medical librarian on December 28, 2022, and rerun on February 22, 2024, using the following bibliographic databases from inception: PubMed/MEDLINE, Cochrane Library (Wiley), and Scopus (Elsevier). The search consisted of a combination of subject headings and keywords including, but not limited to spinal and epidural injections, sciatica, radiculopathy, back pain, intervertebral disc displacement and degeneration, spinal stenosis, spondylosis, prognosis, and predictors. The full PubMed search strategy is provided in Appendix 1. No language, publication date, or study type restrictions were included in the search. Forward and backward citation searching for included articles was completed as well.

### Study selection

**Fig. 1:**
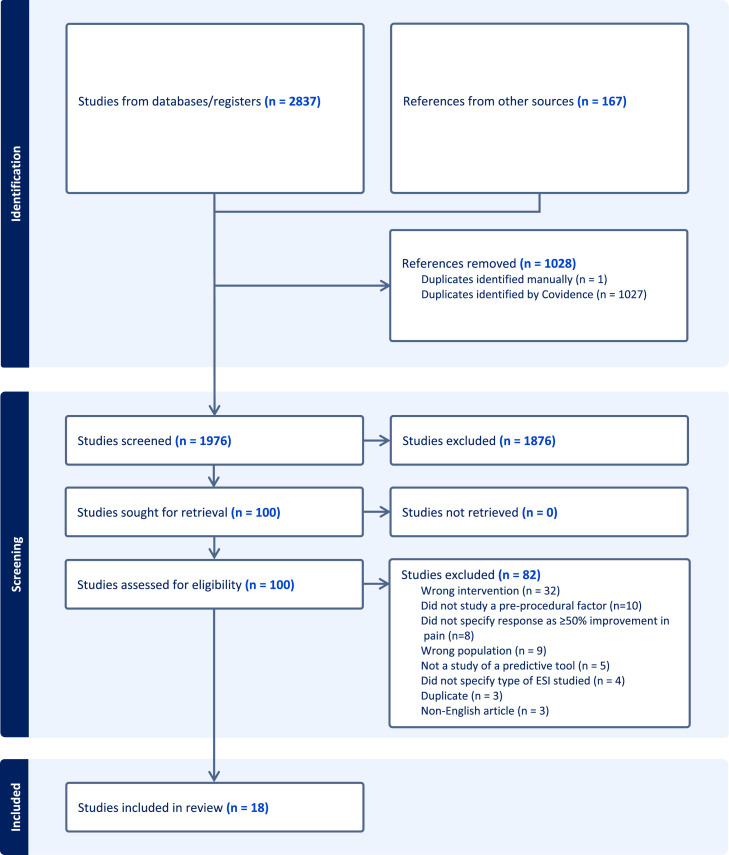
PRISMA diagram outlining search strategy.

The 3,004 results produced from the database searches were imported into Covidence, a systematic review screening tool, and de-duplicated. The remaining 1,976 citations were screened by title and abstract against predetermined inclusion and exclusion criteria. To be eligible, articles had to meet the following inclusion criteria: (1) the study must include adult participants with unilateral or bilateral radicular pain or radiculopathy; (2) participants must undergo a lumbar transforaminal epidural steroid injection using image guidance (computed tomography or fluoroscopy) with contrast medium; and (3) the study must look at a specific prognostic factor for outcomes. Exclusion criteria included (1) studies that included lumbar interlaminar or caudal epidural steroid injections; (2) studies including patients with only axial back pain and no radicular symptoms; (3) studies where patients had prior lumbar spine surgery; (4) studies that did not define or meet success criteria of ≥50% improvement in pain; (5) studies that evaluated procedural or post-procedural factors; (6) systematic or other review types; and (7) articles in a language other than English. One hundred articles were selected for full-text review, 18 of which met inclusion criteria for this study. The PRISMA flow diagram outlining the study selection process was displayed in Fig. 1.

### Data extraction

Two reviewers (MF and SL) extracted data for all the studies which was cross-checked by the research team. A third and fourth reviewer (ES and MR) verified the data. The following information was extracted to describe the variations of studies: study design and setting, sample size and participant sex and age, follow up interval, primary outcome, definition of response, secondary outcome, study conclusion, variable studied, method of assessment, medication injected and method of imaging guidance, levels injected, and number of injections given, method of statistical analysis, and effects of injections. One author (M.F.) initially screened and abstracted articles meeting the inclusion criteria. A second reviewer (S.L.) then independently verified the accuracy of the abstracted data. Any discrepancies identified during the second review were resolved through discussion among the authors.

### Methodological quality

**Fig. 2:**
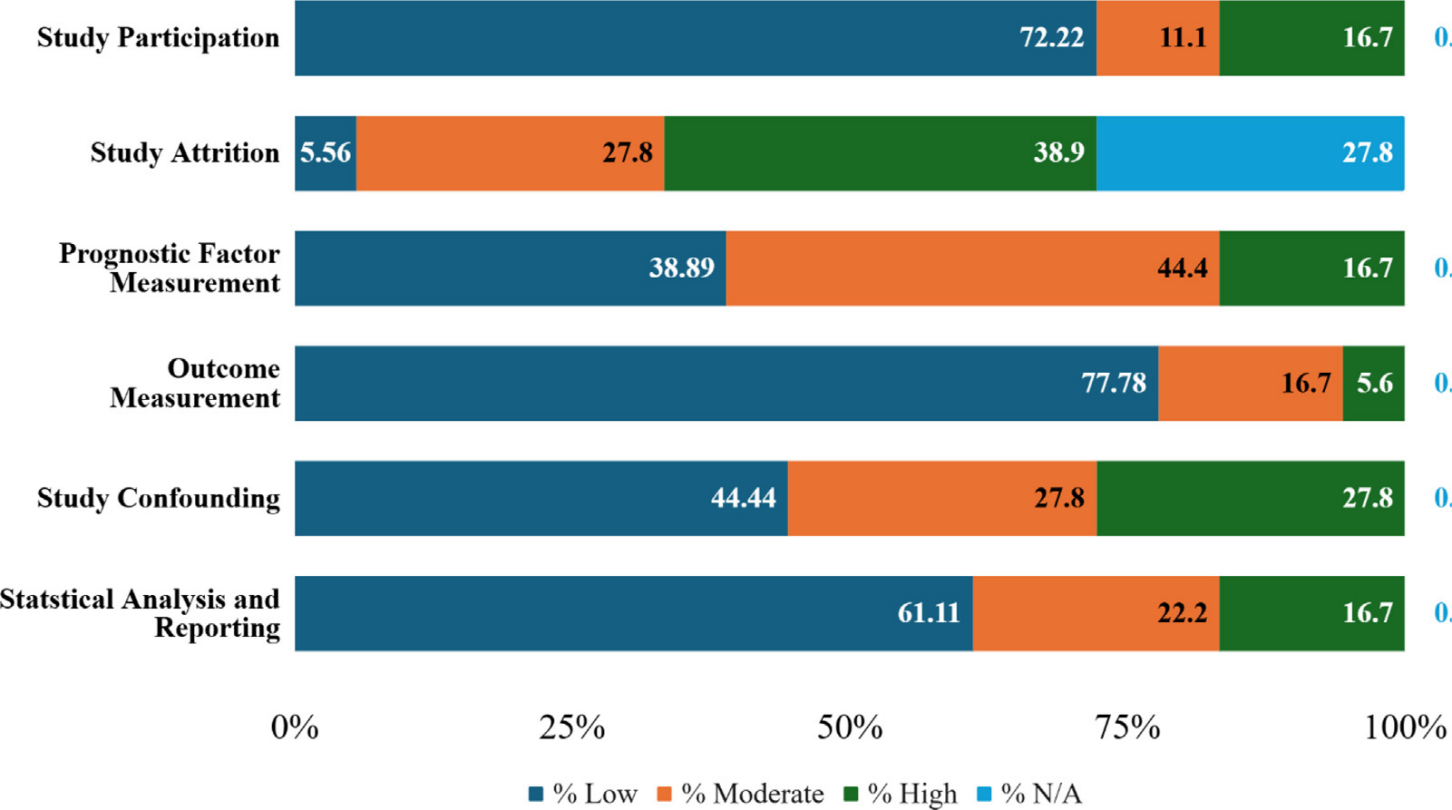
QUIPS risk of bias tool summary based on percentages across studies.

The Quality in Prognostic Studies (QUIPS) tool was used independently by 2 authors (MF and SL) to assess the risk of bias of each study [10]. This tool assesses 6 areas: study participation, study attrition, prognostic factors measurement, outcome measurement, study confounding, and statistical analysis and reporting. The rating for each area was determined to be low, moderate, high or not applicable (Fig. 2, Table 1, Table 2, Table 3, Table 4). If discrepancies in the ratings were observed, the research team reviewed the study and reconciled the differences.

**Table 1:** Summary of study characteristics.

Author	Study design and setting	Sample size and participants. age (years): Mean ± standard deviation (SD), percentage female	Cause of radicular pain	Follow-up (hours/weeks/months/year) in addition to baseline	Primary outcome	Definition of response	Secondary outcome	Conclusion
Adkeniz Leblebicier et al. [11]	Prospective Single center	[46] Mean Age 44±10.8 47% Female	Unilateral L5 root compression confirmed by physical exam, EMG and lumbar MRI, clinical exam. Cause of compression not specified but lumbar spinal stenosis was excluded	1 h, 3 weeks, 3 months	VAS	Two parameters for a successful response were studied: 1) a decrease of ≥80% in VAS and 2) a decrease of ≥80% in VAS and did not undergo surgery after the injection	ODI BDI	The clinical findings and presence of acute involvement on EMG were not predictive for TFESI results; however, the patients with lower scores for the L5 segment in paraspinal mapping benefited more from the injection, compared to patients with higher scores for the L5 segment.
Bahar-Ozdemir et al. [20]	Prospective Single center	[103] Mean age: 48.93±13.39 53% Female	LDH	1 h 3 weeks 3 months	NRS	≥50% NRS reduction Change in ODI score from baseline	ODI HADS SSAS	High preinjection depression scores correlate with decreased pain reduction after LTFESI. High preinjection anxiety and somatization scores do not negatively affect outcomes. Patients with high anxiety and somatization levels had increased preinjection disability scores.
Budrovac et al. [21]	Prospective Study Single center	[59] Median age: 49 years (age range 26 to 65 years) 67.8% female	Single level LDH	Baseline 1 month 3 months	VAS	≥50% reduction in VAS score or reduction by 3 in the VAS compared to the initial value	ODI	LTFESIs help patients with lumbar radicular pain caused by disc herniations with or without disc and nerve root contact, without significant differences.
Celenlioglu, et al. [27]	Prospective Single center	[100] Mean age: 44.8 years 58% Female	LDH	1 h 3 weeks 3 months	NRS	≥50% NRS reduction at 3 months	Modified ODI BDI	Facet tropism correlates with a lower success rate after a LTFESI.
Chang et al. [22]	Prospective Observational, single center	[57] Age range 20 - 79 years old	Lumbar foraminal stenosis	1 month 2 months 3 months	NRS	≥50% NRS reduction at 3 months compared to baseline	Global perceived effect by using a 7-point Likert Scale	LTFESIs reduce pain in lumbar foraminal spinal stenosis of all degrees, but superior results are in patients with mild to moderate lumbar foraminal stenosis than severe lumbar foraminal stenosis
Choi et al. [12]	Retrospective	[68] Age range 13-78 years old 45% Female	LDH with nerve root compression	Follow up range: 7 days-24 months, Average follow up 3.6 months	VAS	≥50% VAS reduction and patient satisfaction score >2 at last visit	Patient Satisfaction scale	Better outcomes were associated with a centrally located disc herniation, and a lower grade of nerve root compression, grade 1 vs. grade 2 or 3.
Cyteval et al. [13]	Prospective case series Single center	[229] Age range 20-85 Mean age: 55 48% Female	1)LDH, 2) other fixed degenerative causes including hypertrophic OA of the zygapophyseal joint, posterior vertebral body osteophytes, or 3)a combination of 1 and 2	2 weeks 1 year	VAS	≥75% excellent, 50-74% good 25-49% fair, ≤25% poor	None	Decreased duration of symptoms before injection correlates with efficacy. Age, level of pain, cause of pain comparing disc herniation vs. spinal stenotic lesion, conflict location, and pain intensity were not predictive factors of radicular pain relief.
Dhandapani et al. [23]	Prospective, single center	[52] Age range 18-65 Mean age, 43.22 years 48.08% Female	Single level LDH	24 h 1 month 3 months 6 months	ODI	Decrease of ODI by 40%, pain reduction >50% on NRS at least 1 month after TFESI	NRS	Patients with 2AB type intervertebral disc protrusions (on the Michigan State University grade profile), demonstrated a lower response rate
Ghahreman et al. [8]	Retrospective of a prospective, randomized trial 2 teaching hospitals	[71] Mean age: 48.2 46% Female	LDH	1 month	VAS	≥50% VAS reduction	SF-36 Roland -Morris Patient-Specified Functional Outcomes Scale Use of other health care Improvements in ADLS	Patients with low grade nerve root compression respond more favorably than high grade nerve root compression. There was no association between response to treatment and location, morphology, dimensions, and size of disc herniation, or associated degenerative changes.
Kim JK et al. [31]	Retrospective Single center	[503] Mean Age: 59.2±14.4 55% Female	LDH and lumbar spinal stenosis	2 months	NRS	≥50% NRS reduction at 2 mo	None	Convolutional neural network deep learning model may be helpful for determining therapeutic outcomes after TFESI for chronic lumbosacral radicular pain
JW Lee et al. [14]	Retrospective Single center	[149] Mean Age: 51.5 49.7% Female	LDH	2 weeks 2 years	Visual analogue scale (VAS)	Very best Outcome: (1) initial response with much improvement or no pain and a reduction in the VAS score of more than 50% on chart documentation 2 weeks after a series of ESI; and (2) more than 2 years’ symptom improvement after ESI without operation. Very worst Outcome: (1) no improvement or aggravated state or a reduction in the VAS of less than 50% on chart documentation after a series of ESI; or (2) temporary improvement after a series of ESI but symptom recurrence in less than 2 months; or (3) temporary improvement after series of ESI but symptom recurrence resulting in operation in less than 6 months.	5-point self-satisfaction scale (no pain, much improved, slightly improved, no change, aggravated)	LDH in either or both the foraminal or extraforaminal zone is the only good MR-based outcome predictor of TFESI for lumbar radiculopathy. Presence of herniated Intervertebral disc zone, T2-high signal, relation to nerve root, corner change, Modic changes, disc height loss, grade of disc degeneration and osteophytes, gender, symptom duration, pattern of symptom attack were not predictive.
JW Lee et al. [15]	Retrospective Single center	[56] Mean age: 53.3 years 58.9% female	Did not specify, “clear identification of the affected nerve root by clinical findings and imaging studies”	2 weeks	VAS 5-point Likert self-satisfaction scale	Patient satisfaction score of ≥3, ≥50% pain reduction	N/A	Transforaminal epidural steroid is an effective tool for managing sciatica, and an extra-epineural injection may be a predictor of a better outcome for sciatica treated using transforaminal epidural steroid. Other predictors of a better outcome include saddle-type contrast distribution pattern, herniated disk, and <6 months of symptoms.
Maus et al. [24]	Retrospective observational study	[516] Mean age: 62.9±15.4 years 57.6% female	LDH, fixed central canal, lateral recess or foraminal stenosis, a combination of fixed stenosis and disc herniation, synovial cysts, epidural fibrosis, or no discernable neural compression	2 weeks and 2 months	≥%0% relief Numerical Rating Scale (NRS) of pain Roland –Morris disability questionnaire (R-M)	Percent change in NRS or R-M from baseline to 2 months post-procedure: ≥50% improvement for NRS, or ≥40% improvement for R-M).	N/A	In the entire sample, outcomes for disc herniations were more favorable than for fixed lesions. However, fixed lesions treated with dexamethasone had outcomes indistinguishable from disc herniations. Single lesions had better out-comes than tandem lesions.
McCormick et al. [16]	Retrospective cohort study. Multi-Center	[188] Mean age: 57.5±17.5 58.0% female	Disc pathology (disc protrusion, extrusion, sequestration, or disc bulges with annular tear), central stenosis, foraminal stenosis, spondylolisthesis, and other	Variable follow up intervals, mean time to follow up was 20.0±14.2 days	Visual analog scale (VAS)	Patients were further grouped into no pain relief or worsened pain (0%), pain relief but <50% relief (>0%*e*<50%), or significant pain relief (50%) on the VAS.	McGill Pain Inventory	Greater baseline pain on the VAS and McGill Pain Inventory, history of a lack of worsening pain with walking, and physical examination findings of a positive femoral stretch test predict more frequent pain reduction after TFESI for lumbosacral radicular pain at short-term follow-up. Greater baseline pain on the McGill Pain Inventory and a lack of worsening pain with walking predict a magnitude of >50% pain reduction.
Sariyildiz et al. [17]	Prospective Single center	[286] Age (18-68) 49% female	LDH	Baseline 3 months	VAS	≥50% reduction in VAS scores	ODI HADS	Shorter duration of symptoms, low grade nerve root compression, foraminal/extraforaminal location on MRI findings were associated with a favorable response.
Sencan et al. [26]	Prospective study Single-center	[64] Median age 42(35-49) in the group with sacralization Median age 47 (39-50.5) in the group without sacralization 53% female	Paracentral LDH at L5-S1 and unilateral L5 or S1 nerve root compression	1 h 3 weeks 3 months	Numeric rating scale	≥50% reduction in NRS scores	Modified Oswestry Disability Index	Sacralization is a risk factor in reducing treatment success after a TFESI.
Sencan et al. [18]	Retrospective study Single-center	[219] Mean age: 43.65±12.18 years 53.9% female	LDH	1 h, 3 weeks, and 3 months	Numerical rating scale (NRS)	≥50% reduction in pain score at 3 months	N/A	A decreased pain score at one hour is a predictor for a favorable 3-month response to TFESI and patients with LDH-induced sciatica.
Tecer et al. [25]	Retrospective observational study Single-center	[59] Mean age: 44±12 years 39.0% female	LDH with or without nerve root impingement	2 weeks and 3 months	Visual analogue scale (VAS)	Improvement in pain by 30 points or by 50%	N/A	TFESI was an effective treatment. method in patients with radicular pain, irrespective of the type or location of disc herniation. However, greater improvement in pain may be expected at the second week in patients with high intensity zone and at the third month in patients with nerve root impingement.

Abbreviations: BDI = Becker depression inventory, BMI = body mass index, EMG = electromyography, HADS = hospital anxiety and depression scale, HIVD = herniated intervertebral disc, HIZ = High Intensity Zone, HRqoL = health related quality of life, LDH = lumbar disc herniation,

LSTV = lumbosacral transitional vertebrae, LTFESI = lumbar transforaminal epidural steroid injection, NRS = numeric rating scale, ODI = oswestry disability index, PGIC = Patient's global impression of change, R-M score = Roland Morris Disability score, SF-12 = 12- item short form survey, SLRT = straight leg raise test, SNRB = Selective Nerve Root Blocks, SSAS = Somatosensory Amplification Scale, VAS = visual analogue scale, VRS = verbal rating scale.

**Table 2:** Factors examined as predictors for lumbar transforaminal epidural steroid injections for radicular pain.

Author	Variable	Method of assessment	Medication injected/method of imaging guidance	Levels injected and number of injections	Method of analysis	Results
Akdeniz Leblecier et al. [11]	Baseline ODI, BDI, paraspinal mapping score, VAS before injection, VAS difference before injection and at 1st hour, SLRT, age, and BMI	EMG collected prior to injection. Patient surveys for pain and function.	Fluoroscopic guidance, 0.5-1 mL contrast, 80 mg methylprednisolone acetate, 1 mL 0.5% bupivacaine, 1 mL saline.	Single-level injection at L5	Shapiro-Wilk test, student's t-test, Mann-Whitney U test, Fisher's Exact test, Chi-Square, Yates's correction, Spearman's rank correlation test.	In comparing patients who achieved *a*≥80% decrease in VAS versus those who did not, there was no significant difference in the preinjection ODI and BDI. The L5 paraspinal score for the patients who had a 3-month VAS decline of ≥80% was significantly lower (*r*=−0.67). In comparing the group who responded negatively to the injection and subsequently underwent surgery, there was no significant differences in age or BMI between these groups (p>.05) The paraspinal mapping score and the L5 paraspinal mapping score were significantly higher in the patients who underwent surgery after the LTFESI (p<.01). Patients who had a positive SLRT from 20 to 40 degrees all underwent surgery, whereas if the SLRT was negative or positive at 40 or higher, the patients did not undergo surgery after the LTFESI (p<.001). Preinjection BDI and ODI scores in patients undergoing surgery were significantly higher than those who did not have surgery after the LTFESI (p<.05 and p<.01).
Bahar-Ozdemir et al. [20]	The role of preinjection depression, anxiety, and somatization on outcomes of the injection	Validated questionnaires	C-arm fluoroscopy and contrast 1 level injection-2 mL of 40 mg/mL methyl prednisolone acetate, 1 mL saline and 1 ml 0.5% bupivacaine, 2 level injection-2 mL of 40 mg/mL methyl prednisolone acetate, 1 mL of saline and 1 mL of 0.5% bupivacaine is divided to each nerve root	1 or 2 levels, unilateral or bilateral	Data distribution analysis: Histogram, Normality plots, Shapiro-Wilk normality test Comparison between 2 groups: For normally distributed parameters: T test, Pearson correlation coefficient for non- normally distributed parameters: Mann-Whitney test, Spearman rank correlation coefficient Statistical Significance: p<.05	Negative correlation between depression level and percent reduction in NRS scores at the 3rd week (*r*=−0.182, p=.022) and third month (*r*=−0.204, p=.037), but not between anxiety and somatization levels and NRS scores. No statistically significant correlation between HADS score and ODI change at pretreatment, 3 weeks, and 3 months (p>.05). Positive correlation between preinjection ODI and HADs-anxiety score (*r*=0.271, p=.001) and SSAS scores *r*=−0.201, p=.013)
Budrovac et al. [21]	To determine the effectiveness of a TFESI depending on whether there is contact between the disc and the nerve root.	Clinical assessment, follow up method not specified.	Fluoroscopic guidance Nonionic contrast 5 mL of 0.25% percent levo-bupivacaine and 40 mg of methylprednisolone	1 level	Absolute and relative frequencies, Two tailed t- tests, Fisher's exact test, McNemar-Bowker test, marginal homogeneity tests, Shapiro wilk test, Mann- Whitney U test, Friedman test, Spearman's correlation coefficient	In the group with disc herniation and nerve root contact, the only significant reduction was in pain intensity (p<.001), but not in the ODI at 1 month (p=.05) and 3 months (p=.25). In the group with disc herniation *without* nerve root contact, there was significant improvement in the pain intensity (p<.001) and ODI after 1 month (p<001) and 3 months (p=.001).
Celenlioglu, et al. [27]	The impact of facet tropism (asymmetry of sagittal orientation of facet joints greater than 10 degrees) on outcomes of injection	Clinical examination	C-arm fluoroscopy and contrast medium 80 mg methylprednisolone acetate, 1cc saline 1cc 0.5% bupivacaine	1 level	Categorical variables: Chi-squared, Fisher's exact tests Nonsequential Categorical Variables: Cochrane Q analysis, Paired t-test Repeated measures in ordinal data: Friedman test Statistical Significance: p<.05 Variance analysis	At week 3 and month 3, the change in NRS and ODI scores was lower in the group with facet tropism than the group without (p<.001). No statistically significant difference in ODI scores for both groups No significant difference between the time-dependent changes of the NRS, ODI, and BDI scores among the patients with LDH at the same level as facet tropism and at different levels
Chang et al. [22]	Severity of lumbar foraminal stenosis (Mild to moderate LFSS vs. severe LFSS) as a predictive factor for outcomes.	Assessments performed by one investigator who was blinded to the group allocation and did not participate in treatment. Grade of lumbar foraminal stenosis determined by lumbar sagittal MRI and findings	C-arm fluoroscopy and contrast medium 20 mg triamcinolone, 0.5 mL bupivacaine (Bupivacaine strength not specified)	1 level	Mann-Whitney U test, Chi squared test, repeated measure one-factor and 2 factor analysis, Bonferroni correction. Statistical Significance: p<.05	Group *A*=mild and moderate lumbar foraminal spinal stenosis Group *B*=severe lumbar foraminal spinal stenosis In both groups, the NRS scores at 1, 2, and 3 months were significantly decreased when compared to pretreatment NRS scores (p=.000). Rates of successful pain relief at 3 months were different between the 2 groups (p=.000). Reductions in NRS scores were larger in group A compared with group B, at 1 month (p=.044), 2 months (p=.021) and 3 months p=.017), and rates of patient satisfaction were significantly higher in group A, compared with group B (p=.000).
Choi et al. [12]	MRI findings -type, location, size of herniation, grade of nerve root compression, presence of spinal stenosis, and hydration. Age, sex, injection level, or preinjection symptom duration were evaluated as predictors.	Clinical evaluation and follow up. Evaluation followed recommendation of the combined task forces of the North American Spine Society, the American Society of Spine Radiology and the American Society of Neuroradiology	C-arm fluoroscopy and contrast medium 0.5% 40 mg triamcinolone, 0.5 cc bupivacaine.	1 level, but repeat injections were performed during the study interval (up to 5 times)	Chi-Square or Fisher's exact tests, Student's T test, Logistic regression analysis	The location of the herniated disc and grade of nerve root compression were different between the responders and nonresponders, p<.05. A centrally located herniated disc was more common in the responder group, and extraforaminal disc herniation was successfully treated. Subarticular disc herniations showed unsatisfactory results. Grade 2 or 3 nerve root compression showed more unsatisfactory results than grade 1 nerve compression (odds ratio: 7.43 and 25.9). There was no significant difference between the responders and nonresponders in terms of the type, hydration and size of the HIVD, or association with spinal stenosis. The injection level, age, sex or the preinjection symptom duration also were not predictors (p>.05)
Cyteval et al. [13]	To identify factors that predict efficacy or failure of LTFESI for nonsurgical sciatic pain. Evaluated duration of symptoms before injection, VAS score before the procedure, age, level, location of LDH, and pain intensity were evaluated.	Initial clinical evaluation with follow up method not specified.	Fluoroscopic guidance and Omnipaque 240 contrast 2 mL of 80 mg of methylprednisolone acetate, 3 mL of 2% lidocaine	1 level	Wilcoxon signed rank test, Fisher exact test, Kruskal Wallis test (measure radicular pain relief categorized in four classes (excellent, good, fair, poor) related to the duration of symptoms before infiltration, intensity of pain prior to injection)	Symptom duration before the procedure was highly correlated with the pain relief outcome. The mean duration of symptoms prior to the injection was 3.04 months (SD 3.28) in the group with excellent relief versus 7.96 months (SD 9.04) in the group with poor pain relief at 2 weeks. The mean VAS scores were 6.5 (range, 3.1–9.5) before and 4.2 (range, 0 –9.5) 2 weeks after the procedure (35.4%), with significant pain relief (p<.001). Age, level, location of LDH, and pain intensity before the procedure were not predictive of outcomes. Statistical significance not reported.
Dhandapani et al. [23]	Studied the grade of lumbar disc protrusion using the Michigan State University grade profile (type 1a, 1 B, 2A, 2 B, 2 AB, 3A) to compare outcomes after LTFESI	Verbal history, assessed in the outpatient dept, review of indoor case sheets	C-arm fluoroscopy, 1 mL nonionic contrast iohexol, 2 mg dexamethasone and 2 mL of 0.25% bupivacaine	1 level	Continuous data analyzed using mean and standard deviation, categorical variables were assessed with the chi-square test, and comparisons of mean scores before, after, and at different injection intervals were conducted using Student's *t*=test, with statistical significant at p<.0001	Patients with 2AB type intervertebral disc protrusions required surgery, which corresponded to a lower response rate, statistical significance not reported.
Ghahreman et al. [19]	Various predictors were studied: Clinical parameters-duration of symptoms and abnormal neurologic exam. Imaging-herniation location, morphology and grade of disc displacement, foraminal narrowing, presence of degenerative changes at the level treated.	Clinical assessment, MRI films were reviewed independently by a specialist neurosurgeon and a pain specialist, each blinded.	PA Fluoroscopy with contrast medium 0.75 mL 0.5% bupivacaine, 70 mg triamcinolone	1 or 2 levels	Continuous data: Two-sample t-test Categorical data: contingency tables, chi-squared test. Strength of positive associations were assessed by calculating sensitivity, specificity, and positive likelihood ratio Method of Cohen	74% of patients with low-grade nerve compression responded favorably to treatment, whereas 26% of those with high-grade nerve root compression responded (p<.000). There was no association between response to treatment and any of the other clinical variables examined: Duration of symptoms (p=.979), presence of neurologic exam findings (p=.413 and p=.692), herniation location (p=.164, .217, .293), morphology of herniation (p=.346, .290, .542), and grade of disc displacement (p=.959, .057, .859, .105), presence of degenerative changes at the level treated (p=.266).
Kim JK et al. [31]	Using machine learning, specifically a trained CNN (Convolutional neural network) deep learning model using whole lumbar spine sagittal T2-weighted MR images to predict outcomes of TFESI due to HLD or lumbar spinal stenosis	Chart review and all injections performed by a single interventional physiatrist	C-arm Fluoroscopy with 0.3 mL of nonionic contrast material to check vascular uptake, 20 mg, triamcinolone and 0.5 mL bupivacaine and 1 mL normal saline.	1 level	Python 3.8.8 and Sckikit-Learn version 0.24.1, 95% confidence interval for the AUC was described by DeLong et al.	Validation accuracy was 76.2%, AUC was 0.827 Training accuracy was 75.9%, the AUC was 0.839
JW Lee et al. [14]	MR-based outcome predictors were studied including the HIVD level, HIVD type, HIVD zone, HIVD volume, T2 signal in the HIVD, HIVD-nerve root relationship, Modic changes, disc height loss, grade of disc degeneration, and presence of posterior osteophytes. Age, sex, symptom duration, previous symptom attacks were studied.	Chart review by a radiologist, MRI findings evaluated by 2 blinded radiologists per ASSR, ASNR, and NASS guidance.	Fluoroscopic guidance, contrast (iohexol 300 mg I/mL), 40 mg triamcinolone, 1 mL 0.5% bupivacaine, 0.5 mL saline	Series of 1-3 injections, 2 weeks apart for repeat injections. Single-level injections. Levels L2/3, L3/4, L4/5, L5/S1.	Chi-square tests, Mann-Whitney U test, Fisher's Exact Test	HIVD in the foraminal–extraforaminal zone was significantly more common in the very best outcome group than HIVD in the central–subarticular zone (p=.012). In the age group of 60-69 years, patients in the very best outcome group were more common than those in the very worst outcome group (p<.007). Other factors such as the HIVD level (p=.278), HIVD type (p=.393), HIVD volume (p=.299), T2-signal in the HIVD (p=.380), HIVD-nerve root relationship (p=.544), presence of Modic changes (p=.339), disc height loss (p=.762), grade of disc degeneration (p=.677), and presence of posterior osteophytes (p=1.000) were not statistically significant. Sex (p=.508), symptom duration(p=.235), previous symptom attack (p=.166) were also not predictive of outcomes.
Lee et al. [15]	Age, sex, and duration of sciatica before injection, Intra-epineural versus extra-epineural contrast pattern, saddle-type versus non-saddle-type contrast distribution pattern, spinal stenosis vs herniated disk being the cause of radicular pain,	Spot radiographs to document contrast material distribution, as assessed by 3 MSK radiologists. Intra/extra-epineural injections via Pfirrmann Classification. Chart review.	Fluoroscopic guidance, 1 mL iohexol, 300 mg I/mL contrast, bupivacaine (0.5 mL / 0.5%); 40 mg (1 mL) triamcinolone acetonide suspension.	1-level injection. Levels included L1-S1.	Fisher's exact test, chi-square test, multiple logistic regression analysis.	Intra-epineural injection was effective in 19 patients (65.5%) and the extra-epineural injection in 24 (88.9%) patients. An extra-epineural injection was associated with a better outcome (p=.04, odds ratio:5.01). The following were not statistically significant predictors: saddle-type versus non-saddle-type contrast distribution pattern(p=.5), cause of sciatica between spinal stenosis vs herniated disk, (p=.35), age (p=.2), sex(p=.55), and duration of sciatica (< 6 months vs > 6 months) (p=.28).
Maus et al. [24]	Imaging characteristics of compressive lesions Steroid formulation (particulate or nonparticulate) and tandem lesion as prognostic factors were evaluated.	Most recent preprocedure CT/CT myelogram/ MRI reviewed by certified neuroradiologists and categorized as follows: 1) disc herniation, 2) fixed lesion, 3) combination of fixed lesion and disc herniation, 4) synovial cyst, 5) combination of disc and synovial cyst, 6) combination of fixed lesion and synovial cyst, 7) epidural fibrosis, 8) no compressive lesion.	Injection performed per SIS guidelines. Used betamethasone, triamcinolone or dexamethasone	1 level injection. Levels not specified.	Chi-Squared tests and t-tests, Multivariable logistic regression models.	Patients with disc herniations had greater functional recovery than those with fixed lesions (p=.006). Single compressive lesions have better pain outcomes than tandem compressive lesions (p=.029) In patients with fixed lesions, nonparticulate steroids, dexamethasone, had a higher proportion of responders than the particulate steroids (p=.01). Patients with grade 3 compression (neural displacement with obliterated fat/CSF) had the greater proportion of responders, followed by grade 2,4, and 1 (p=.048).
McCormick et al. [16]	Demographic, clinical, and radiologic factors specifically: age, sex, smoking status, employment status, worker's compensation benefits, duration of pain at presentation, elements of the history, physical exam findings, radiologic findings.	Clinical History. Physical Exam: Femoral stretch test for patients receiving TFESI at L2-3, L3-4, L4-5, slump sit test and SLR for patients getting injections at L4-5, L5-S1 and S1. *Imaging. VAS/McGill Pain Questionnaires*	Fluoroscopic guidance, 0.2-0.5 mL iopamidol contrast dye, 1.5-2 mL 1% lidocaine, 1-2 mL betamethasone 6mg/mL or triamcinolone 40mg/mL injected, volume dependent on 1-2 level or uni- vs. bilateral procedure. All subjects received the same total steroid dose.	1 or 2 levels (1-level unilateral, 1-level bilateral, 2-level unilateral). Levels L2-3, L3-4, L4-5, L5-S1, S1 foramen.	Bivariate associations using Chi-square tests, Kruskal-Wallis test.	Greater baseline pain on the VAS (p=.0001) and McGill Pain Inventory (p=.0358), history of a lack of worsening pain with walking(p=.0161), and physical examination findings of a positive femoral stretch test (p=.0477) predict more frequent pain reduction after TFESI at short-term follow-up. These factors failed to provide predictive value: Demographics and history: age (p=.5534), sex (p=.7662), smoking status (p=.1212), employment status (p=.8902), worker's compensation status=.6839), duration of pain at presentation p=.4200), baseline Pain Anxiety Symptoms Scale 20 score(p=.3645), baseline 10-item Center for Epidemiologic Studies-Depression score(p=.8681) baseline ODI score (p=.2585) Exam: pain worse with sitting(p=.4309), better with sitting (p=.7274), worse with bending forward (p=.0892), worse with standing(p=.5379),worse with lying(p=.3809),better with lying (p=.4379), worse with physical therapy(p=1.000), worse with lifting (p=.1810), better with walking (p=.0822), provocation of pain with seated slump (p=.9911), straight leg raise (p=.2659), strength deficit on physical examination(p=.5451) Radiologic findings: discogenic pathology(p=.8182), presence of central stenosis(p=.0985), presence of foraminal stenosis (p=.7343), spondylolisthesis (p=.5954).
Sariyildiz et al. [17]	Age, BMI, symptom duration, and MRI characteristics including the location, type of disc bulge, muscle weakness, and depression, anxiety	Method of follow up not specified	C-arm fluoroscopic guidance, 0.5-1 mL Iohexol, 1.5 mL of 40 mg triamcinolone acetonide, 2% lidocaine, 2.5 mL saline	1 level. If a patient had inadequate relief at 2 weeks, an additional 1-level TFESI was administered.	Kolmogorov-Smirnov and Shapiro-Wilk tests for normally distributed variables, nonparametric tests to analyze non-normally distributed variables, continuity correction chi-square test of Fisher's exact test for categorical data, Student t-test to evaluate pre and post TFESI application differences within groups, Pearson's or Spearman's correlation analyses, p<.05 and 95% confidence interval were considered statistically significant.	Correlation analyses revealed that shorter duration of symptoms before injection (p=.009), low grade nerve root compression (p<.001) and a foraminal/extraforaminal location on MRI were associated with a favorable response. Multi-variate logistic regression analysis revealed that patients with higher nerve root compression, Pfirrmann Grade 3, had worse outcomes (pain and functional status) than patients with Grades 1 and 2 (p<.001). The following were not predictive of the outcome: Age (p=.736), BMI (p=.714), LDH location (p=.084), LDH morphology (p=.234), muscle weakness (p=.207), HADS depression score (p=.412) and HADS anxiety score (p=.359).
Sencan, et al. [26]	Presence of LSTV, specifically sacralization, excluded lumbarization)	A physiatrist determined the presence of sacralization and category of sacralization using Castellvi classification via MRI and AP lumbar X-ray. NRS scores and MODI questionnaires completed by subjects.	Fluoroscopic guidance, 1-2cc contrast, 1cc 0.5% bupivacaine, 1cc saline, 4 mg betamethasone.	1-level injection. Levels L5 or S1.	Pearson's chi square test, Friedman test and post hoc Dunn test, Mann- Whitney U test.	NRS and modified ODI scores improved at 3 weeks and 3 months in both groups with or without sacralization (p<.05). At 3 months, NRS scores were higher in the group with sacralization than without (p=.026). NRS and disability scores were higher in type 3B LSTV and disability scores higher in the type 2B LSTV group at week 3 (p<.05).
Sencan et al. [18]	Evaluated whether a decreased pain score at 1 h is a predictor of a favorable 3-month response to LTFESI in patients with LDH induced sciatica. Patient characteristics including age, sex, body mass index (BMI), duration of symptoms, presence of transitional vertebrae, grade of nerve compression and injection levels were also studied.	Chart review.	Fluoroscopic guidance, 1-2 mL of contrast dye, mixture of 80 mg methylprednisolone acetate, 1cc physiological saline, 1cc 0.5% bupivacaine.	1-level injection. Levels L4-L5, L5-S1, or S1 foramen	Shapiro-Wilk test, Mann-Whitney U test, Chi-Square test, Multivariate regression analysis, univariate and multivariate analyses.	Decreased pain scores at 1 h was a predictor of treatment success at 3 months(p=.024). Age (p=.616), sex(p=.383), BMI(p=.776), injection level (p=.508), transitional vertebrae (p=.554), duration of symptoms(p=.051), or grade of nerve root compression (p=.803) was not predictive of outcome.
Tecer et al. [25]	MRI findings including LDH type (bulging, protrusion, extrusion), location (central, subarticular, foraminal, extraforaminal), the presence of a high intensity zone (HIZ), and the presence or absence of nerve root impingement.	Chart review with MRI imaging. VAS prior to injection and at 2 weeks and 3 months after.	Fluoroscopic guidance, Omnipaque 300 contrast agent, 1 mL betamethasone acetate 3mg/mL and betamethasone sodium phosphate 3.947 mg/mL with 1 mL 2% lidocaine	1-level injection. Levels L3-S1.	Friedman statistic, Mann-Whitney U test, Wilcoxon signed rank test.	Pain scores improved significantly in each group (p<.05) at all time points, but there were no statistically significant differences in improvements according to type or location of disc herniation. Pain scores were significantly lower in patients with a HIZ at the 2nd week and in patients with nerve root impingement at the third month (p<.05).

**Table 3:** Prognostic factors studied.

Prognostic factor category	Prognostic factors/[# of studies]	Studies that evaluated the prognostic factors
Patient characteristics	Age, [8]	[[11], [12], [13], [14], [15], [16], [17], [18]]
	Symptom duration prior to injection [8]	[[12], [13], [14], [15], [16], [17], [18], [19]]
	Sex, [5]	[12,[14], [15], [16],^18^]
	Psychiatric comorbidities [3]	[16,17, 20]
	BMI [3]	[11,17, 18]
	Preinjection pain intensity [2]	[13,16]
	Level of pain [2]	[12,18]
	Preinjection ODI/functional status [1]	[11]
	Smoking status [1]	[16]
	Employment type/status [1]	[16]
	Worker's Compensation status [1]	[16]
Physical exam findings	Neurological testing [3]	[11,16, 19]
	Motor weakness [2]	[16,17]
Radiologic characteristics	MRI findings-Lumbar disc herniation characteristics-location, type, size, hydration, presence or absence of disc contact with nerve root, degree of disc contact with nerve root Lumbar spinal stenosis characteristics-presence and degree of neuroforaminal stenosis, canal dimensions [13]	[[12], [13], [14], [15], [16], [17], [18], [19],[21], [22], [23], [24], [25]]
	Single/tandem lesion [1]	[24]
	Presence of high intensity zone (HIZ) [1]	[25]
	Deep learning using T2 sagittal MRI images [1]	[31]
Anatomical variances	Lumbosacral Transitional Vertebrae [2]	[18,26]
	Facet Tropism [1]	[27]
EMG	Paraspinal mapping score [1]	[11]

**Table 4:** Assessment of domains of potential bias (QUIPS tool).

Author	Study participation	Study attrition	Prognostic factor measurement	Outcome measurement	Study confounding	Statistical analysis and reporting
Akdeniz Leblebicier et al. [11]	Low	Moderate	Moderate	Low	Moderate	Low
Bahar-Ozdemir et al. [20]	Low	Moderate	Low	Low	High	Moderate
Budrovac et al. [21]	Low	Moderate	Moderate	Low	Low	Low
Celenlioglu et al. [27]	Low	Moderate	Moderate	Low	Low	Low
Chang et al. [22]	Low	Moderate	Low	Low	High	Low
Choi et al. [12]	Low	N/A	Low	Low	Moderate	Low
Cyteval et al. [13]	Low	High	High	Low	Moderate	Moderate
Dhandapani et al. [23]	Low	High	Moderate	Low	High	High
Ghahreman et al. [19]	High	N/A	High	Moderate	Moderate	Low
Kim JK et al. [31]	Low	High	High	Moderate	High	High
JW Lee et al. [14]	Low	N/A	Low	Low	Low	Low
JW Lee et al. [15]	Moderate	Low	Moderate	Low	Low	Low
Maus et al. [24]	High	High	Moderate	Low	Low	Moderate
McCormick et al. [16]	Moderate	N/A	Moderate	Moderate	Low	Low
Sariyildiz et al. [17]	Low	High	Low	Low	Low	Low
Sencan et al. [26]	Low	High	Low	Low	Low	Low
Sencan et al. [18]	Low	High	Moderate	High	High	High
Tecer et al. [25]	High	N/A	Low	Low	Moderate	Moderate

## Results

There were 9 prospective studies and 9 retrospective studies included. Overall, 20 prognostic factors were evaluated in 5 broad categories across 18 articles, with many studies evaluating multiple prognostic factors. The most frequently evaluated predictive factors were the MRI findings contributing to radicular pain, including characteristics of lumbar disc herniations and degree of nerve root compression (*n*=13). Age (*n*=8), symptom duration prior to the injection (*n*=8), and sex (*n*=5) were the next most frequently evaluated predictive factors for outcomes. The studies were published between 2006 and 2023 with a total of 2,825 participants. The follow-up in the studies ranged from 2 weeks to 2 years, with a median follow-up of 3 months, and a mean follow-up of 5.38 months. All injections were given via fluoroscopic guidance (*n*=18). The steroid injected included triamcinolone (*n*=7), methylprednisolone (*n*=6), dexamethasone (*n*=1), and betamethasone (*n*=2), with 2 studies using multiple steroids or an unspecified steroid. Regarding local anesthetic, bupivacaine (*n*=13), and lidocaine (*n*=4) were used. One study did not specify local anesthetic usage or type. Injection volumes (including saline) ranged from 1 to 5 mL of injectate.

### Patient characteristics

In the vast majority of studies, age [[11], [12], [13], [14], [15], [16], [17], [18]], sex [12,[14], [15], [16],^18^], BMI [11,17,18], smoking status [16], employment status [16], and worker's compensation status] [16] were not found to be predictive of outcomes. In one study, a lower preinjection functional status [11], though not associated with pain relief was a predictive factor for those who went on to have surgery. Two studies found that a shorter duration of symptoms was predictive of better pain relief from a LTFESI [13,17] while others found no associations [12,[14], [15], [16],^18^,^19^]. In one study, preinjection pain intensity was not associated with outcomes [13] while another showed that greater baseline pain was predictive of pain reduction at short term follow up [16]. The level of pain was not found to be correlated to pain relief [12,18]. Among studies where psychiatric comorbidities were evaluated, 2 studies found no associations with improved pain [16,17]. One study found that depression was correlated with less pain reduction after injection while anxiety and somatization did not have a relation to improved outcomes after injections [20].

### Clinical findings

Neurological testing and physical exam findings [11,16,19] were evaluated and found to have mixed findings. In one study, patients who had decreased pain relief and underwent surgery after a LTFESI, had a straight leg raise test (SLRT) which positive at 30-40 degrees, while patients who were most likely to respond to the LTFESI had a SLRT which was negative or positive at 40 degrees or higher [11]. In another study, a positive femoral stretch test, which is used for detecting L2-4 nerve root injury, was predictive of pain relief after the injection [16]. One study found no correlation between neurological exam findings and pain relief [19]. Having strength deficits on physical examination did not correlate with outcomes [16,17].

### Imaging

The largest predictive factor studied were the structural pathologies causing radicular pain and their relationship to outcomes, including the characteristics of LDHs (lumbar disc herniations) and grade of nerve root impingement as measured on the MRI [12,13,[15], [16], [17], [18], [19],[21], [22], [23], [24]]. Four studies showed that the location of the LDH had no association with the improved pain [13,17,18,25] while a positive response was associated with central disc herniation [12], and a foraminal and extraforaminal LDHs in [14,17]. The type of LDH [12,14,19,25] and size of the LDH [12,14,19] was not found to be related to improved pain. Four studies showed lower grade nerve compression had better results [12,17,19,22], while 1 study showed that higher nerve compression had favorable results [24]and 1 study showed no relation to outcome [18]. One study specifically evaluated patients with radicular pain who had a disc herniation with and without nerve root contact. It was found that patients with nerve root contact improved in pain intensity but not in function, but the group who had a herniation without nerve root contact improved both in pain and function [21]. Most articles found no significant difference when degenerative changes were associated with the LDH [12,14,16,19]. Two studies found no difference when the cause of radicular pain was from a lumbar disc herniation or spinal stenosis [15,16].

### Anatomical variants

Two studies reviewed the presence of lumbosacral transitional vertebrae (LSTV) [18,26]. One study found no predictive value of LSTV on results [18], whereas one study concluded that the presence of sacralization was found to have reduced pain relief in the group with sacralization at 3 months, and reduced pain and functional improvement in outcomes after a LTFESI in Type 2B and Type 2B in the Castellvi classification at 3 weeks, but no difference at 3 months [26]. One article found that facet tropism defined by authors as an asymmetrical alignment of same level facet joints greater than 10° as a negative predictive factor for improvement from LTFESI [27].

### EMG

Lower paraspinal mapping scores at the L5 segment correlated with better pain outcomes than higher scores. Additionally, higher paraspinal mapping scores were associated with patients who underwent surgery after the LTFESI. The presence of acute involvement on EMG did not affect results [11].

## Discussion

This was the first systematic review to provide an in-depth summary of recent literature that evaluated preprocedural prognostic factors and their relationship to outcomes after LTFESIs. It is not surprising that the most studied areas were regarding the MRI findings causing radicular pain, given that the 2012 clinical guidelines from the North American Spine Society give a Grade A recommendation for MRI as a diagnostic study to evaluate lumbar disc herniation with radiculopathy. We show that not only are the structural pathologies seen through MRI findings helpful for diagnosis, but they can also be predictive in identifying outcomes after a LTFESI.

This study confirmed findings in one systematic review [28] that also showed an association of low-grade nerve root compression on lumbar MRI as predictive of short-term pain reduction after an epidural steroid injection, although that study included lumbar interlaminar steroid injections as well as cervical epidural steroid injections. A more recent systematic review [29] included injections performed via the transforaminal, interlaminar and caudal routes. They also found better outcomes with a lower grade of nerve root compression. This is likely related to the fact that severe lumbar neuroforaminal stenosis is often accompanied by morphologic changes of the nerve root on lumbar MRI, which can cause chronic mechanical stimulation of nociceptive nerves as well repeated inflammation [22] which may explain reduced effects from a steroid injection. The authors also postulate that severe neuroforaminal stenosis can impede anterior epidural flow during the injection procedure, which may limit the quantity of steroid that reaches the nerve root. Regarding other prognostic factors, we came to slightly different conclusions from the previous review in that they found that there was a poorer outcome in the setting of depression and a LSTV, while our study found articles that conflicted those studies so that the same conclusion could not be made. Their study found an association with improved outcomes if the disc herniation was central or nonforaminal, while our study found that central, foraminal, and extra-foraminal herniations were all associated with positive outcomes. If a disc is not in a subarticular location, there is an association with favorable outcomes after LTFESIs.

This review found that outcomes were not affected if degenerative changes accompanied a LDH as the cause of radicular pain or if the cause of the radicular pain was from a disc herniation or from lumbar spinal stenosis. This informs clinical practice in that patients with radicular pain can still benefit from the procedure regardless of the cause, whether the pain is from an acute or chronic cause, or a combination.

This review included a single study that showed worse outcomes in the setting of facet tropism at the level of nerve root compression. This was a novel article in that facet tropism is not commonly considered as a factor that would affect outcomes of an epidural steroid injection but can easily be identified on axial imaging of a CT scan or MRI. Further study of this predictive factor is needed but its potential association as a prognosticating factor is promising.

## Strengths and limitations

### Strengths

A strength of this review is that it focuses on predictive factors specific to LTFESIs rather than a combined study of all epidural approaches. Since the efficacies of the different approaches (interlaminar, caudal, transforaminal) can have different success rates inherent to what part of the epidural space the steroid is placed, studying prognostic factors specific to LTFESIs helps clinicians make more relevant recommendations when suggesting this treatment to patients. The inclusion criteria covered a wide range of preprocedural prognostic factors that were evaluated among a variety of causes of radicular pain, including LDH and degenerative changes, or a combination of both, allowing a comprehensive search that also mimics the varied patient presentation in a typical spine practice. Including all causes of radicular pain allowed for the comparison of whether degenerative changes affect outcomes and whether acute or chronic causes of radicular pain were associated with different outcomes after a LTFESI, and we found out that outcomes were not affected. Many articles included many secondary prognostic factors beyond the primary factor(s). Including all the secondary factors enhanced the thoroughness and robustness of the review. In addition, specifying a minimal clinically important difference as ≥50% pain relief allowed for the selection of studies that were more relevant to the outcomes measured in clinical practice.

### Limitations

Although the inclusion and exclusion criteria narrowed the review to patients who had similar symptom presentation with a similar route of injection treatment, there was inherent variability in the included articles regarding the type and dose of steroids that were used. As there is wide variability in the type and amount of steroid that is used in clinical practice [30], a systematic review that limited criteria to a single type or dose of steroid would narrow the scope of the review especially since it is unclear whether a difference in outcome can be attributed to a specific type or dose of steroid.

The follow up period in the studied articles was variable, with 5 out of the 18 studies having patients followed for less than 3 months. However, many studies followed patients even further than 3 months with a mean follow-up of 5.38 months. Another limitation could be that the study included injections performed at all levels of the lumbosacral spine from L1 to S1. We felt that narrowing the review to articles that studied injections at the same level in the lumbar spine would be restrictive, but this could be an area of further study.

## Conclusions

This systematic review has identified many prognostic factors associated with outcomes of LTFESI for radicular pain. Among the most studied factors, the MRI characteristics including the degree of nerve root compression and the location of the herniation, are significant. This review shows that patients have improved outcomes when there is a lower grade of nerve compression and if the lumbar disc herniation is not in a subarticular location but in the central, foraminal or extraforaminal space. There is improvement with a LTFESI for radicular pain whether the LDH is accompanied by degenerative changes and whether the radicular pain is from a disc herniation or from lumbar spinal stenosis. Lower paraspinal mapping scores on EMG are correlated with better outcomes than higher scores. Most studies show no association of outcomes with age, sex, BMI, smoking status, employment status, workman's compensation status, type and size of the disc herniation, or acute findings on electromyography. There are mixed findings on whether psychiatric comorbidities correlated with pain reduction and whether the presence of a lumbosacral transitional vertebrae affect results. To enhance the robustness of future studies, a more uniform approach in terms of standardized outcome measures and longer follow-up periods is recommended. Larger prospective prognostic studies are needed to confirm findings especially when findings were mixed or when only a single study explored the prognostic variable. In addition, exploration of procedural factors or even post-procedural outcomes to predict longer term outcomes would also be a valuable area of study.

### Implications

The evidence behind factors that predict outcomes from a LTFESI have varying degrees of bias, but trends appear that can be used to guide patient expectations and clinical decision making. Patients with a lower grade of nerve compression with a disc that is not in a subarticular location have better outcomes. Patients who have lower paraspinal mapping scores, indicating milder lumbar radiculopathy, have better outcomes than those with a higher paraspinal mapping score. Outcomes are not affected if a lumbar disc herniation is accompanied by degenerative changes or if the cause of radicular pain is from a lumbar disc herniation or from lumbar spinal stenosis. The factors that have been identified with improved outcomes can be applied in the clinic to guide patient expectations on whether a LTFESI is likely to relieve their radicular pain or consider transitioning from interventional spine treatment earlier in favor of a more definitive treatment option, such as surgery.

## Declaration of competing interests

The authors declare that they have no known competing financial interests or personal relationships that could have appeared to influence the work reported in this paper.

## Appendix 1

PubMed search strategy

("Injections, Spinal"[Mesh] OR "Spinal injection*"[tiab] OR "spine injection*"[tiab] OR "Injections, Epidural"[Mesh] OR "selective nerve root block*"[tiab] OR "epidural injection*"[tiab] OR "Transforaminal"[tiab] OR "Steroid Injection*"[tiab])

AND

("Sciatica"[Mesh] OR "Sciatica"[tiab] OR "Radicular pain"[tiab] OR "Radicular lumbar pain"[tiab] OR "Radicular low back pain" [tiab] OR "Radiculopathy"[Mesh] OR Radiculopath*[tiab] OR "Neuropathic pain" [tiab] OR "Spinal pain"[tiab] OR "spine pain"[tiab] OR "Nerve impingement"[tiab] OR "Nerve Root Compression*"[tiab] OR "Lower extremity pain"[tiab] OR "Leg Pain"[tiab] OR "Low Back Pain"[Mesh] OR "Back Pain"[Mesh] OR "back pain"[tiab] OR "backache"[tiab])

AND

("Intervertebral Disc Displacement"[Mesh] OR "intervertebral disk hernia"[tiab] OR "Herniated Intervertebral Disc"[tiab] OR "Herniated lumbar disc"[tiab] OR "Herniated disc"[tiab] OR "Disc herniation" [tiab] OR "Disc prolapse"[tiab] OR "Spinal Stenosis"[Mesh] OR "Spinal stenosis"[tiab] OR "foraminal stenosis"[tiab] OR "central stenosis"[tiab] OR "canal stenosis"[tiab] OR "subarticular stenosis" [tiab] OR "acquired stenosis"[tiab] OR "degenerative spin*"[tiab] OR "Osteoarthritis, Spine"[Mesh] OR "Intervertebral Disc Degeneration"[Mesh] OR "intervertebral disk degeneration"[tiab] OR "lumbar degenerative disc disease"[tiab] OR "Spondylosis"[Mesh] OR "spondylosis"[tiab])

AND

(prognos*[tiab] OR Predict*[tiab] OR Efficacy[tiab] OR effect*[tiab] OR affect[tiab] OR success[tiab] OR outcome[tiab] OR relation*[tiab] OR associat*[tiab])

NOT surgery[ti]

NOT fusion[ti]

NOT discectomy[ti]

NOT cervical[ti]
